# A Behavioral Approach to the Tourism Consumer Decisions of Generation Z

**DOI:** 10.3390/bs12090320

**Published:** 2022-09-04

**Authors:** Silvia Puiu, Liliana Velea, Mihaela Tinca Udristioiu, Alessandro Gallo

**Affiliations:** 1 Department of Management, Marketing and Business Administration, Faculty of Economics and Business Administration, University of Craiova, 200585 Craiova, Romania; 2 Department of Humanities, University Ca’Foscari, 30123 Venice, Italy; 3 Department of Physics, Faculty of Sciences, University of Craiova, 200585 Craiova, Romania

**Keywords:** sustainable tourism decision, healthy behavior, waste reduction, Generation Z, hospitality industry

## Abstract

The main objective of our research is to identify the impact of recycling and waste reduction behavior on the sustainable tourism decisions of Romanian youngsters (18–25 years old). We used the PLS-SEM method and introduced four variables in the model: sustainable tourism decisions, the interest in recycling, the interest in waste reduction, and the interest in natural and less polluted touristic destinations. The main results emphasize the direct influence of recycling and waste reduction behaviors on the decisions made by Generation Z regarding sustainable tourism and on their preference for destinations that are better preserved and less touched by human intervention. The novelty of our research consists of the fact that we introduced variables such as waste reduction from the perspective of tourists because most studies address it as a management approach of the companies in the tourism sector. The findings are useful for managers in the tourism sector to create better strategies for attracting the younger generation who are preoccupied by environmental issues and sustainability in general.

## 1. Introduction

Sustainability is a topic of interest for governments, citizens, and companies that want to ensure that future generations will not be affected by current economic development and consumption habits. Tourism as an important sector for many countries can impact the environment, contributing to climate change, an increased carbon footprint, and the deterioration of natural resources. For this reason, tourism should become more sustainable, and this is the responsibility not only of the public authorities and organizations in this sector, but also of all of us as citizens and tourists.

According to UNEP and UNWTO [1] (p. 11), sustainable tourism “is based on the principles of sustainable development” referring to all three pillars of sustainability, which are economic, social, and environment. The report emphasizes that these principles should be applied to all types of tourism. There are also a few goals in the 2030 Agenda where sustainable tourism can be included: goal 8 referring to a sustainable economic growth, goal 9 referring to a sustainable industrialization, goal 11 referring to sustainable cities and communities, goal 12 referring to sustainable consumption and production, goal 14 referring to life below water, and goal 15 to life on land. All of these goals could be targeted and reached by the companies in the tourism sector in order to develop more sustainable tourism.

According to Santos-Roldán et al. [2] (p. 1), sustainable tourism is “a source of healthy tourism”, which implies that this should be the normality in this sector. Creaco and Querini [3] also highlight the importance of the tourism sector for a country’s sustainable development. According to Eurostat [4], the tourism industry registered 2.3 million of entities and 12 million employees in 2018 at the level of EU-27, almost 10% of the total non-financial businesses. This contribution is significant, which highlights the fact that promoting more sustainable tourism would bring important benefits for the development of a country and the entire region, ensuring a healthier environment for future generations.

The present research focuses on sustainable tourism from the perspective of youngsters representing Generation Z. The age group 18–25 years old is specific to people at the beginning of their adult life, who have just finished their education and are starting their careers. This generation is important because their habits, including those related to touristic preferences, can influence future generations too. Also called the Digital Generation, Generation Z representatives are more preoccupied by the environment and sustainability in general [5,6,7]. As Wood [6] (p. 2) states, youngsters in this generation are “more scarcity-oriented” and pay attention to the way “they spend their money”. The author also mentions the education received in families and the current context in which environmental issues are more debated in media. Hysa et al. [7] mention the financial restrictions and limited budgets of this generation at the beginning of their careers, and that sometimes this translates into more sustainable decisions. Haddouche and Salomone [8] (p. 77) name these youngsters “tomorrow’s travellers”, which makes it important for managers in the tourism sector to better understand this generation.

## 2. Literature Review and Hypotheses Development

The present study analyzes the impact of recycling and waste reduction behaviors on the decisions for practicing more sustainable tourism among Generation Z. Thus, we used four variables in accordance with the latest studies in this field: sustainable tourism decisions, the interest in recycling, the interest in waste reduction, and the interest in natural and less polluted touristic destinations.

### 2.1. Sustainable Tourism Decisions

Generally, this concept refers to the decisions made by authorities, communities, companies, and tourists when engaging in touristic activities. Our research focuses on the perspective of young tourists and their decisions when travelling to various locations. Miller [9] highlights the role played by consumers in the way tourism is carried out. For more sustainable tourism and for healthier decisions, companies should better address the needs of a generation that is more environmentally aware with regard to the impact of their decisions.

Pomering et al. [10] have a marketing approach on sustainable tourism that brings the focus closer to the tourist. Garg and Pandey [11] analyzed factors such as tourists’ knowledge and their perception of how effective their decisions are on the sustainability of tourism. Their results show that knowledge on these issues increases the chances of adopting more sustainable behaviors when travelling. Wehrli et al. [12] present the problem of sustainable tourism decision from the marketing perspective and the way companies in the tourism industry should promote their products and services in order to ensure that tourists will choose more wisely. Bausch et al. [13] point out the cultural differences between tourists in different countries in the way they understand sustainable tourism as a concept and the way they understand how to apply it when travelling. Thus, the authors emphasize the important role of marketing campaigns that can help tourists in making more informed, sustainable, as well as healthier decisions for themselves, for the environment, and for the community in general.

### 2.2. The Interest in Recycling 

This variable was chosen for the purpose of our research taking into account the interest of the younger generation in protecting the environment and thus recycling more frequently. Chaturvedi et al. [14], in their study conducted in a developing economy, state that the preoccupation of Generation Z with the environment influences their decision to buy recycled clothes. Wang et al. [15] conducted a study on Generation Z in China to analyze their behavior regarding plastic recycling. The authors emphasize that this young generation is “more concerned with the environment”.

Miller et al. [16] mention the habit of recycling, which means that tourists who are already accustomed to recycling, and thus protect the environment, will continue this healthy behavior during their vacations. The authors highlight the importance of creating a proper infrastructure in touristic locations to encourage and promote these eco-friendly behaviors among tourists and residents.

Grazzini et al. [17] use the term “activate” to show that hotel managers should implement better strategies to raise the awareness of visitors and make them recycle more frequently. Juvan and Dolnicar [18] (p. 42) have a similar perspective, considering that hotels can deliberately “activate the guests’ pro-environmental intentions and lead to the intended behaviour”. Firth and Hing [19] present examples of accommodation units that are more eco-friendly and thus encourage tourists to adopt greener behavior, with recycling included.

In other words, tourists with an interest in recycling can motive managers in touristic locations to invest in the infrastructure and create better conditions for recycling, but the opposite is also true. Hotel managers can encourage or spark the interest in recycling if they make the facilities friendlier, thus the recycling becomes easier and more accessible for everyone.

### 2.3. The Interest in Waste Reduction 

MacInnes et al. [20], as well as Miller et al. [16], mention that tourists’ habits to protect the environment continue during their vacations. Thus, waste reduction is a habit that cannot be stopped. The authors [20] (p. 2) analyze behaviors related to “shower duration” (water waste), “reusing towels, eating up all the food ordered … waiving unnecessary routine room cleaning at the hotel, and returning/refusing single-use products”. Their finding that habit plays such an important role could be helpful for educational managers and public authorities in creating campaigns that help with the formation of such healthy behaviors.

A study of Dolnicar et al. [21] reveals that reducing the food waste in hotels by reducing the plate does not affect tourists’ satisfaction, but reduces the cost for the accommodation unit and the carbon footprint. This means that waste reduction can be stimulated among tourists, but also that tourists, through their habitual behaviors, can stimulate hotel managers to adopt strategies in this direction. Whitmarsh et al. [22] point out the consistency in the habit to reduce waste and recycle and conclude that this behavior is also maintained during holidays, but to a lesser extent than at home.

### 2.4. The Interest in Natural and Less Polluted Touristic Destinations

There are several studies that emphasize the role played by the level of pollution and the natural beauty of a destination in tourists’ preferences. Rodrigues et al. [23] highlight the importance of air quality for a touristic location, pointing out that most visitors pay attention to this aspect. This finding is encouraging because it might determine authorities and organizations to implement strategies in the direction of reducing pollution, thus taking steps towards more sustainable tourism. Xue and Gao [24] (p. 1) consider “air pollution as a travel constraint” and a compromise that tourists make when they cannot avoid pollution. According to Chhetri et al. [25], the natural beauty of touristic destinations enhances tourists’ satisfaction. MacKay and Fesenmaier [26] appreciate that the perception regarding the beauty of a touristic decision is also dependent on cultural factors. Kim et al. [27] state that beautiful landscapes are preferred by Chinese tourists. Regarding air pollution, things are clearer because people want to breath fresh air and enjoy their vacation, but related to beauty, some tourists might appreciate natural landscapes, while others prefer the vibe of a crowded city. Urban or city tourism is also emphasized in many works [28,29,30].

### 2.5. Hypotheses Development

Considering the increased interest in sustainability in tourism and the role it plays in developing economies [31,32,33,34,35,36,37,38], we developed five hypotheses that can be useful for researchers, public decision-makers, and companies in the tourism sector.

**Hypothesis** **1** **(H1).** *The interest in recycling directly and positively influences sustainable tourism decisions.*

**Hypothesis** **2** **(H2).** *The interest in waste reduction directly and positively influences sustainable tourism decisions.*

**Hypothesis** **3** **(H3).** *The interest in natural and less polluted touristic destinations directly and positively influences sustainable tourism decisions.*

**Hypothesis** **4** **(H4).** *The interest in recycling directly and positively influences the interest in natural and less polluted touristic destinations.*

**Hypothesis** **5** **(H5).** *The interest in waste reduction directly and positively influences the interest in natural and less polluted touristic destinations.*

## 3. Research Methodology

**Figure 1:**
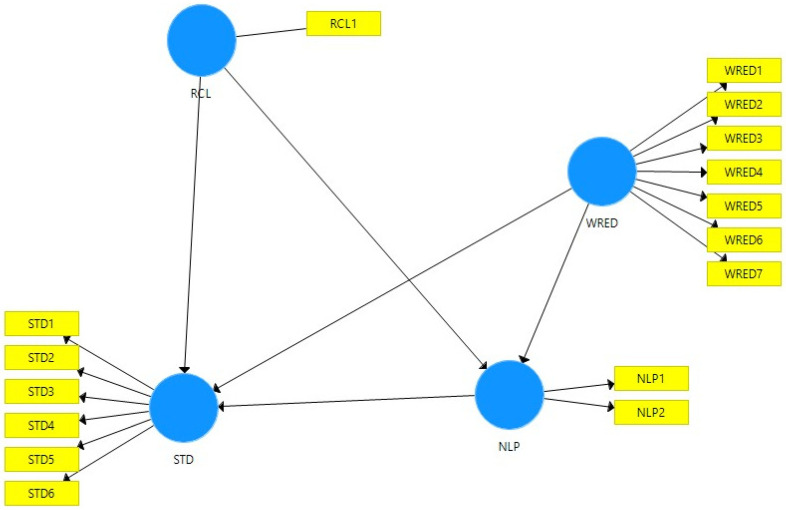
The research model. Source: Created using SmartPLS.

We applied PLS-SEM using SmartPLS v.3 [39], the preferred method for small samples like ours. The model we developed is presented in Figure 1 and includes four variables: sustainable tourism decisions (STD), which has six items (STD1–STD6); the interest in recycling (RCL), which has one item (RCL1); the interest in waste reduction (WRED), which has seven items (WRED1–WRED7); and the interest in natural and less polluted touristic destinations (NLP), which has two items (NLP1 and NLP2).

The model constructs as well as their items and codes are summarized in Table 1. There are also presented in the studies that helped us to formulate the questions in the survey.

We used a five-point Likert scale for the questions in our research, with the respondents choosing their answers on a scale from total disagreement (1) to total agreement (5). The survey created with Google Forms was distributed by e-mail (chosen from an internal database) and social media channels (mainly Facebook, because it is one of the most used channels in Romania) in September and October 2021 to 870 representatives of Generation Z (18–25 years old) from Romania using a non-probability sampling technique. We received 158 valid questionnaires. We chose only respondents in this age group because youngsters under the age of 18 do not make their own decisions when traveling, being minors. The reason for focusing our research on sustainable tourism on Generation Z lies in the latest studies in the field [7,8,53]. This generation is more interested in sustainability when travelling compared with previous generations [53], because of their values, but also because they are facing some financial restrictions and limited budgets, which makes them more minimalist and more creative when planning their vacations.

## 4. Results

To check the model’s convergent validity, we determined the outer loadings for the items included in the model. As we can see in Table 2, most outer loadings are above 0.7, with this value being considered as an indicator for a higher convergent validity [54]. The authors advise undergoing a careful analysis before removing the items with outer loadings below 0.7 from the model. From the five items with outer loadings below the threshold of 0.7 (STD1, STD5, WRED1–WRED3), we decided to keep STD1 and STD5 because of their importance in the model. STD1 refers to more sustainable transportation when travelling and STD5 to restaurants that are more eco-friendly, using vegan/vegetarian options. Besides accommodation, transportation and restaurants are an important part of a vacation, which makes them important for our analysis. Thus, we removed only three items (WRED1–WRED3) with the lowest outer loadings. The VIF values for all items in the model are under 4, which ensures the collinearity statistics. 

**Figure 2:**
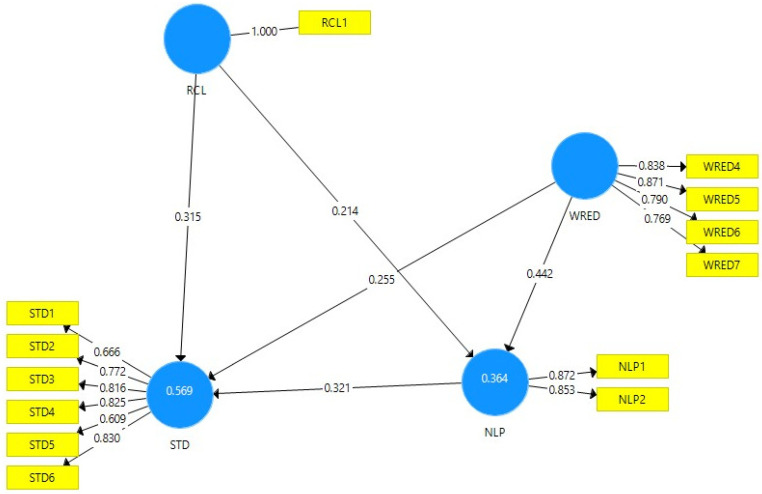
The path coefficients and outer loadings. PLS algorithm calculation. Source: calculated with SmartPLS.

After removing the items with outer loadings below 0.6, the model changes, as we can see in Figure 2. The strongest impact is from WRED to NLP (0.442), followed by NLP to STD (0.321), and from RCL to STD (0.315). The lowest impact is from RCL to NLP (0.214). The positive values show a positive correlation between the variables. RCL, WRED, and NLP account for 56.9% of the STD variance, and RCL and WRED account for 36.4% of the NLP variance, as we can notice from Figure 2. 

In order to determine the construct reliability and validity, Cronbach’s alpha and average variance extracted (AVE) were calculated. The numbers are summarized in Table 3. The values for Cronbach’s alpha are higher than 0.8 for three of the constructs, which indicates a high reliability. For NLP, the Cronbach’s alpha is between 0.6 and 0.7, which is appreciated as acceptable by many authors [55,56,57]. All AVE values are above 0.5 and composite reliability is higher than 0.8 for all four constructs in the model, which shows a high validity and reliability of the variables in the model.

The Fornell–Larcker criterion was used to determine the discriminant validity of the research model. The main diagonal in Table 4 shows the values for AVE’s square roots, which are above the other values in the same column. This certifies the discriminant validity of the constructs included in the research model.

We applied the bootstrapping test in order to check the significance of the model. Thus, t statistics, *p*-values, and the confidence interval bias corrected are summarized in Table 5 for all five connections between the variables. At a 5% significance level, all t statistics are higher than 1.96 and all *p*-values are lower than 0.05, which shows that all model path coefficients are statistically significant. 

We can also notice from Table 5 that none of the confidence intervals bias corrected for the five correlations in the model includes the zero value, which validates all of the hypotheses we formulated. The analysis for the hypotheses’ validation is presented in Table 6.

We applied the blindfolding test to analyze the structural model. As we can notice from Table 7, the Q2 values for NLP (0.257) and STD (0.316) are higher than 0, which confirms a high predictive relevance of the variables in the model.

In Table 8, we summarize the descriptive statistics for all of the items remaining in the model. All means are above 3, with one exception. STD5 has a mean of 2.538 as well as the lowest outer loading (0.609). Taking into account that the item refers to the preference of restaurants offering vegan and vegetarian options, we might say that this is in accordance with the culinary preferences in Romania [58,59].

## 5. Discussion

The hypotheses were all validated, showing that sustainable tourism decisions made by Generation Z in Romania are directly and positively influenced by recycling and waste reduction behaviors. These healthy behaviors also influence the preference for less polluted and more natural touristic destinations.

**H1.***The interest in recycling directly and positively influences sustainable tourism decisions.* The hypothesis is validated, with the result being similar to those of other studies [16,61,62,63,64]. Youngsters accustomed to recycling on an everyday basis will adopt the same behaviors during their vacations, which ensures better and more sustainable decisions. This is an important aspect, but it should be accompanied by similar preoccupations from all tourism actors (public authorities, hotels and other accommodation units, and restaurants).

**H2.***The interest in waste reduction directly and positively influences sustainable tourism decisions.* The hypothesis is validated, in accordance with other studies [20,65,66,67,68]. All of these studies show a correlation between the habit of avoiding waste and a more sustainable form of tourism. Moreover, youngsters in Generation Z are more minimalist, avoiding waste in general, which translates into sustainable behaviors [69].

**H3.***The interest in natural and less polluted touristic destinations directly and positively influences sustainable tourism decisions*. The hypothesis is validated, showing a direct relationship between the interest in natural locations that are better preserved and less affected by pollution and human intervention and, respectively, the decisions made by generation Z towards sustainable tourism. The results are similar to those of other studies in the field [70,71,72,73], which emphasize the role played by tourists’ preferences in ensuring sustainable tourism.

**H4.***The interest in recycling directly and positively influences the interest in natural and less polluted touristic destinations*. This hypothesis is validated, showing that tourists adopting recycling behavior are also more inclined to prefer touristic locations that are cleaner, less polluted, and in a more natural state. The finding is in accordance with other studies in the literature [74,75], which show a direct relationship between recycling behaviors of tourists and the state of the destination (the pollution level and the natural beauty). Wu and Geng [75] also highlight that pollution affects negatively the healthy behaviors of tourists from an environmental perspective.

**H5.***The interest in waste reduction directly and positively influences the interest in natural and less polluted touristic destinations*. This hypothesis is validated, showing that youngsters who are preoccupied with reducing waste are also more interested in visiting destinations that are more natural and with cleaner air. Other authors also addressed this problem of waste management and the preservation of touristic destinations [76,77].

## 6. Conclusions

The main results of our study show that the decisions made by the young tourists in Romania in terms of their recycling and waste reduction behaviors directly influence tourism sustainability. If there are already healthy habits, youngsters will adopt them even during their vacations, thus contributing to the sustainable development of touristic destinations. These findings should make authorities and managers in the tourism sector understand the importance of creating a proper infrastructure that could help tourists to recycle, minimize waste, and be more responsible in general. Hypotheses 3–5 also show that youngsters are more attracted to natural destinations, which are less affected by human intervention. This can be capitalized only if there are adequate means created for proper recycling and waste reduction.

From a theoretical point of view, the results can help researchers as a starting point to analyze the factors that contribute to sustainable tourism decisions for other generations, as well as for comparisons with other countries. The country’s culture and economic development can impact tourists’ behaviors and decisions, as shown by other studies [32,78,79].

The practical implications of our research refer to the usefulness of our findings for the companies in the tourism industry, which can better adjust their strategies in order to promote a more sustainable form of tourism as well as offer a greater experience to tourists. Understanding the preoccupation of Generation Z with sustainability, recycling, and waste reduction, managers in the hospitality sector should invest in adequate infrastructure and tailor their marketing strategies to promote more sustainable tourism and healthier behaviors when visiting touristic destinations. Taking into account that youngsters would pay more for touristic services that are more sustainable (STD6 has a mean of 3.829 and 62.7% of the respondents agreed partially or totally with this statement), hotel and restaurant managers as well as local authorities should more seriously address the problem of sustainability in tourism.

The limits of our research consist of the fact that the questionnaire was distributed online because of the restrictions imposed during the pandemic, and thus some categories of youngsters were not included, such as those with poor or no Internet connection. For future research, we appreciate that more variables could be added to extend the factors affecting the sustainable tourism decisions of Generation Z, such as gender [80], income, and studies. Moreover, a comparison with other generations or other countries could be helpful to better understand how cultural habits and economic development influence the approach to sustainable tourism.

## Figures and Tables

**Table 1:** Constructs and items in the model.

Constructs	Items	Codes	Source
Sustainable tourism decisions (STD)	I prefer to choose less polluting transportation means for traveling.	STD1	[40,41,42,43]
	I prefer to practice a form of tourism that is more friendly with the environment.	STD2	Own contribution
	I prefer eco-friendly accommodation options during my vacation.	STD3	[40,44]
	I come back to accommodation units that are more eco-friendly.	STD4	[44]
	I prefer restaurants which offer vegan and vegetarian menus.	STD5	[45,46]
	I would pay more for a more sustainable vacation.	STD6	[40,44]
The interest in recycling (RCL)	I am interested in recycling even during vacations.	RCL1	[17]
The interest in waste reduction (WRED)	I prefer to use only one set of towels during short stays in an accommodation unit.	WRED1	Own contribution
	I prefer to not use resources offered by the housing unit (cosmetics, hygiene products, bathrobe etc.) if I do not need them.	WRED2	Own contribution
	I prefer to use my own hygienic products in a housing unit to avoid waste.	WRED3	Own contribution
	I avoid water waste during my vacations as much as possible.	WRED4	[47,48] Fortuny et al., 2008; Higgins-Desbiolles, 2018
	I avoid energy waste as much as possible during my vacation.	WRED5	[37,47,48]
	During my vacation, I pay attention to not waste the food at restaurants or all-inclusive accommodation units.	WRED6	[49]
	When buying souvenirs, I prefer sustainable materials to avoid plastic waste.	WRED7	Own contribution
The interest in natural and less polluted touristic destinations (NLP)	I pay attention to the pollution level of the touristic destination I choose.	NLP1	[50,51,52]
	I prefer touristic regions where nature is intact or there are few human interventions.	NLP2	Own contribution

**Table 2:** Outer loadings and VIF values.

Items	Outer Loadings	Collinearity Statistics (VIF)
STD1	0.665	1.735
STD2	0.773	2.017
STD3	0.816	2.088
STD4	0.825	2.129
STD5	0.608	1.403
STD6	0.831	2.168
RCL1	1.000	1.000
WRED1	0.335	1.269
WRED2	0.590	1.791
WRED3	0.553	1.479
WRED4	0.822	3.696
WRED5	0.858	3.989
WRED6	0.770	1.759
WRED7	0.749	1.466
NLP1	0.868	1.313
NLP2	0.857	1.313

Source: Authors’ analysis using SmartPLS.

**Table 3:** Construct reliability and validity.

Construct	Cronbach’s Alpha	rho_A	Composite Reliability	AVE
NLP	0.656	0.658	0.853	0.744
RCL	1.000	1.000	1.000	1.000
STD	0.849	0.864	0.889	0.574
WRED	0.837	0.848	0.890	0.669

Source: Authors’ analysis using SmartPLS.

**Table 4:** Fornell–Larcker criterion.

Construct	NLP	RCL	STD	WRED
NLP	0.863			
RCL	0.502	1.000		
STD	0.627	0.643	0.758	
WRED	0.581	0.652	0.647	0.818

Source: determined using SmartPLS.

**Table 5:** The bootstrapping test.

	T Statistics	*p*-Values	Confidence Interval Bias Corrected
NLP -> STD	3.181	0.002	(0.113, 0.514)
RCL -> NLP	2.125	0.034	(0.012, 0.394)
RCL -> STD	3.904	0.000	(0.141, 0.459)
WRED -> NLP	4.870	0.000	(0.252, 0.611)
WRED -> STD	2.800	0.005	(0.083, 0.428)

Source: calculated using SmartPLS.

**Table 6:** Hypotheses’ validation.

Hypothesis	Validation
RCL -> STD (H1)	Supported
WRED -> STD (H2)	Supported
NLP -> STD (H3)	Supported
RCL -> NLP (H4)	Supported
WRED -> NLP (H5)	Supported

Source: Authors’ analysis.

**Table 7:** The blindfolding test.

Construct	SSO	SSE	Q^2^
NLP	316.000	234.932	0.257
RCL	158.000	158.000	
STD	948.000	648.063	0.316
WRED	632.000	632.000	

Source: calculated using SmartPLS.

**Table 8:** Descriptive statistics for the remaining items.

Items	Mean	Standard Deviation	Outer Loading
NLP1	3.633	1.328	0.872
NLP2	4.171	1.090	0.853
RCL1	3.747	1.316	1.000
STD1	3.684	1.232	0.666
STD2	4.095	1.087	0.772
STD3	3.127	1.339	0.816
STD4	3.671	1.318	0.825
STD5	2.538	1.422	0.609
STD6	3.829	1.190	0.830
WRED4	3.728	1.353	0.838
WRED5	3.867	1.267	0.871
WRED6	4.133	1.179	0.790
WRED7	3.475	1.408	0.769

Source: calculated using JASP [60] and SmartPLS.

## Data Availability

Not applicable.
